# Fibroblast-like Synoviocytes as Key Regulators of Homeostasis and Inflammation in the Joint Microenvironment of Inflammatory Arthritis

**DOI:** 10.3390/biomedicines14020396

**Published:** 2026-02-09

**Authors:** Shih-Ching Lee, Ping-Han Tsai, Tien-Ming Chan, Kuang-Hui Yu

**Affiliations:** 1 Graduate Institute of Biomedical Electronics and Bioinformatics, National Taiwan University, Taipei 106, Taiwan; bioinformair@gmail.com; 2 Division of Rheumatology, Allergy and Immunology, Department of Internal Medicine, Chang Gung Memorial Hospital, Taoyuan 333, Taiwan; 3 Division of Rheumatology, Allergy and Immunology, New Taipei Municipal Tucheng Hospital, New Taipei City 236, Taiwan; 4 Medical College, Chang Gung University, Taoyuan 333, Taiwan

**Keywords:** inflammatory arthritis, FLS, ANGPTL, MMP, FGF

## Abstract

**Background**: The body maintains homeostasis by inflammation, and arthritis is related to autoimmunity or inflammation. Angiogenesis contributes to synovitis through angiogenic factors and proteolytic enzymes, while different inflammatory arthritis conditions, such as osteoarthritis and rheumatoid arthritis, share similar cytokine networks and immune cell populations. Notably, progressive joint damage can occur despite effective systemic immunosuppression, suggesting that local stromal–immune interactions within the joint microenvironment may sustain inflammation and tissue destruction. **Methods**: We conducted an exploratory single-cell RNA-sequencing analysis using publicly available datasets from the NCBI GEO database, including synovial tissue and synovial fluid samples. Cell–cell communication and transcriptional regulatory networks were inferred using CellChat and SCENIC. **Results**: Computational analyses suggested that, in RA, macrophage-associated signaling shifts from TNF-related pathways toward SPP1-associated patterns, coinciding with transcriptional features of MMP3^+^ fibroblast-like synoviocytes (FLS). FLS–FLS interactions were associated with FGF-related signaling across disease contexts. ANGPTL-related signaling patterns differed among arthritis subtypes, with ANGPTL4 more prominent in OA and PsA and ANGPTL2 more frequently in RA-related transcriptional programs. **Conclusions**: These findings provide an exploratory framework for stromal–immune interactions and ANGPTL-associated signaling across inflammatory arthritis. The therapies for PsA may focus on systemic immune modulation and preservation of joint structural integrity. For OA and RA, the highlight may target ANGPTL4 and ANGPTL2 in the early and late stages of disease progression. Given the reliance on computational inference, the results warrant further experimental validation.

## 1. Introduction

Inflammatory response is the primary immunological response of the body to maintain homeostasis after exposure to pathogenic infection, endogenous injury, or tissue stress [1]. Inflammatory arthritis includes a group of arthritis caused by autoimmunity and inflammation of the synovial membrane, accompanied by joint pain, swelling, warmth, tenderness, and morning stiffness in the joints, and even joint damage over time [2]. Angiogenesis, the formation of new capillaries from preexisting vessels, plays a pivotal role in the pathogenesis of synovitis. It is initiated by the release of angiogenic factors that activate endothelial cells, leading to the secretion of proteolytic enzymes such as matrix metalloproteinases (MMPs). These enzymes degrade the basement membrane and perivascular extracellular matrix (ECM), facilitating subsequent endothelial cell proliferation and migration into the perivascular space [3]. Inflammation is associated with angiogenesis of marked changes in the blood vessels on morphologic and histologic examination [4]. Angiogenic factors, such as angiopoietin-like (ANGPTL) proteins, have been implicated in the regulation of angiogenesis and inflammatory processes. A previous study using an adjuvant-induced arthritis model reported that blockade of ANGPTL4 with neutralizing antibody was associated with reduced fibroblast-like synoviocyte (FLS) migration and attenuated inflammation-induced osteoclastogenesis [5].

Rheumatoid arthritis (RA), psoriatic arthritis (PsA), and osteoarthritis (OA) share many similarities, including their potential to cause articular cartilage destruction, synovitis, and osteophyte hyperplasia [6]. In terms of pathogenesis, OA has traditionally been considered to result from mechanical stress, wear, or damage, whereas RA and PsA are regarded as autoimmune diseases [7]. However, certain cytokines such as TNF, IL1β, and IL6, as well as immune cells such as macrophages and T cells, appear to play very similar roles in driving the overall immune response in both conditions [8,9,10,11]. Whether shared stromal signaling programs exist across arthritis subtypes, and how they diverge in disease-specific contexts, remains unclear.

A previous study demonstrated that synovial molecular signatures differ between patients who respond to biologic therapies targeting IL-6 or CD20, with fibroblast-associated gene signatures markedly enriched in non-responders [12]. These observations suggest that FLSs in the joint microenvironment may contribute to persistent joint inflammation through mechanisms that are not fully dependent on canonical immune-inflammatory pathways [13,14].

However, the specific FLSs involved, their functional heterogeneity, and their interactions with immune and stromal cells remain poorly defined. Addressing this gap, our study applies single-cell transcriptomic analysis to systematically characterize synovial cellular heterogeneity and stromal–immune interactions across arthritis subtypes, with a focus on identifying cell populations and signaling pathways that may underlie disease persistence beyond immune suppression.

## 2. Materials and Methods

### 2.1. Single-Cell RNA-Sequencing Dataset

**Figure 1:**
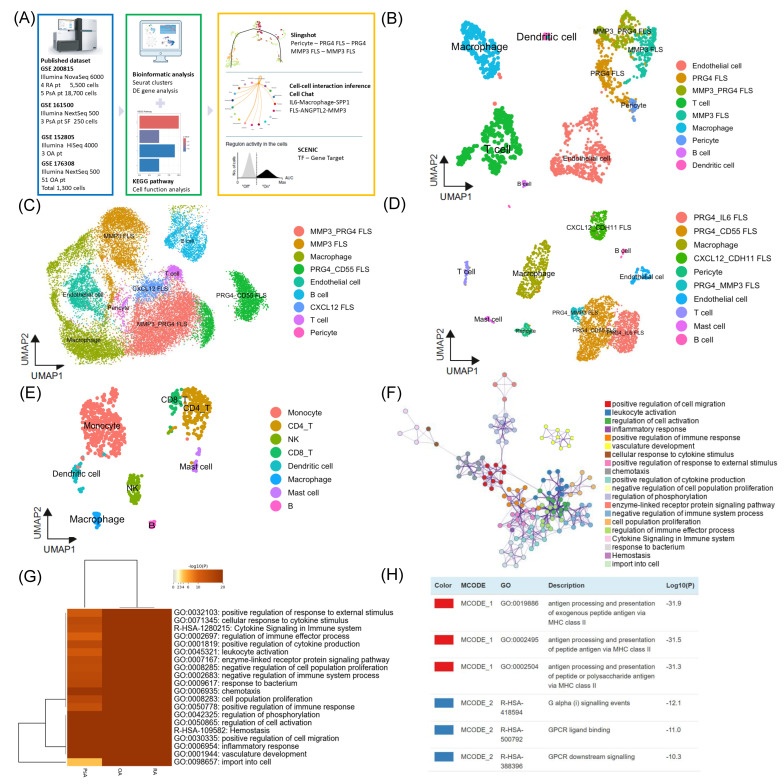
Single-cell RNA-sequencing analysis of synovium and synovial fluid samples from osteoarthritis (OA), rheumatoid arthritis (RA), and psoriatic arthritis (PsA). (**A**) Overview of study design and analysis. (**B**–**E**) Overview of the scRNAseq landscape of GSE159354. Markers were used to identify the clusters and differences and UMAP. (**B**) RA, (**C**) PsA, (D) OA, and (**E**) PsA synovial fluid (**F**) Enriched ontology clusters of all samples. (**G**) Heatmap with GO analysis of all three types of arthritis. (**H**) The MCODE analysis of all three types of arthritis. The protein–protein interaction of MCODE_1 and 2 components.

Single-cell RNA-sequencing (scRNA-seq) datasets were obtained from the NCBI Gene Expression Omnibus (GEO) under accession numbers GSE200815, GSE176308, GSE152805, and GSE161500. All datasets consisted of publicly available, de-identified data in open-access format; therefore, they were exempt from local institutional review board (IRB) review and patient consent requirements in accordance with the Common Rule. Tissue samples in GSE200815, GSE176308, and GSE152805 were derived from synovial tissue biopsies, whereas GSE161500 was obtained from synovial fluid samples. The GSE200815 dataset comprised synovium samples of the knee from four patients with RA and five with PsA. The GSE161500 dataset included synovial fluid samples from the knees of three patients with PsA. The GSE176308 comprised three datasets of pooling synovium samples of 51 knee OA patients. The GSE152805 comprises nine datasets generated from synovium samples from three knee OA patients. Patients were considered biological units. Batch effects related to tissue origin were addressed during data integration. ScRNA-seq was performed using the 10× Genomics Chromium platform, and libraries were sequenced on Illumina HiSeq 4000, NovaSeq 6000, and NextSeq 500 platforms (Figure 1A). Raw reads were processed, assembled, and aligned to the GRCh38 human reference genome using Cell Ranger (v3.1.0, 10× Genomics, Pleasanton, CA, USA).

### 2.2. Data Processing and Analysis, Clustering, and Cell-Type Annotation

Expression count matrices were processed in Seurat (v4.4.0) with cells retained if >500 features and <20% mitochondrial content [15]. Data were log-normalized, and approximately 2000 variable features were selected via mean–variance regression for anchor-based integration and batch correction [15]. PCA was performed, and the top 40 PCs were used for clustering with the Louvain algorithm (resolution = 0.8) on the shared nearest neighbor (SNN) graph. The “Elbow plot” (Figure S1A) was performed using the elbow function, which is a ranking of principal components (PCs) based on the percentage of variance. An ‘elbow’ was observed around PC 39–40, suggesting that the majority of the true signal was captured in the first 40 PCs. Batch effects were subsequently corrected using the Harmony algorithm implemented in the Harmony R package (v1.2.4). The Harmony-corrected embeddings were used for downstream dimensionality reduction, clustering, and visualization. Uniform manifold approximation and projection (UMAP) was applied for visualization. Differentially expressed genes were identified using the FindAllMarkers function in Seurat with thresholds set to |avg_log2FC| > 1 and adjusted *p*-value < 0.05, and clusters were annotated using the CellMarker 2.0 database (http://www.bio-bigdata.center/)(accessed on 27 January 2025) [16].

### 2.3. Functional Enrichment Analysis

For enrichment network visualization and MCODE component analysis, the Metascape [17] and Cytoscape [18] were conducted (http://metascape.org/) (accessed on 6 February 2025). Subsequently, differentially expressed genes (DEGs) were further analyzed for Gene Ontology (GO) and Kyoto Encyclopedia of Genes and Genomes (KEGG) pathway analyses to identify key signaling pathways executed by specific cell populations using the ClusterProfiler package (v4.12.6) [19].

### 2.4. Pseudotime Trajectory Analysis

The trajectory (pseudotime) analysis was conducted by Slingshot (v2.12.0) [20], with UMAP visualization as input and a designated cell cluster as the starting point. Slingshot infers lineage hierarchies from preexisting clusters and orders of cells along pseudotime trajectories.

### 2.5. Cell–Cell Communication Analysis by Ligand Receptor Interaction

Cell–cell communication analysis was performed using CellChat (v2.1.2) [21], based on ligand–receptor interactions inferred from single-cell transcriptomic data of synovial tissue and fluid samples from patients with RA, PsA, and knee OA.

### 2.6. Protein Interaction Network Analysis

The protein–protein interaction (PPI) network was analyzed from the STRING database (version 12.0) (https://string-db.org/) [22]. Nodes represent proteins, and edges represent known or predicted functional associations based on experimental data, curated databases, co-expression, and text mining evidence. The predicted functional partner genes selected by high-confidence interaction scores (>0.7) were applied to minimize false-positive associations. We retrieved the network clustering and pathway enrichment analysis from ANGPTL2 and ANGPTL4.

Gene Ontology (GO) biological process enrichment analysis was performed using STRING by statistically assessing the overrepresentation of annotated GO terms among the input protein list relative to a background set. Enrichment significance was evaluated using a hypergeometric test with Benjamini–Hochberg false discovery rate correction. GO terms with adjusted *p* < 0.05 were considered significant.

### 2.7. Transcription Factor Regulatory Network Analysis

We applied SCENIC (v1.3.1) to predict transcription factor (TF) regulatory networks [23]. In the SCENIC workflow, GENIE3 is to identify potential TF-targets based on coexpression. The RcisTarget is to perform the TF-motif enrichment analysis and identify the direct targets (regulons). The area under the curve (AUC) values are normalized into normalized enrichment scores (NES). Motifs with higher NES indicate regulatory activity over a larger proportion of top-ranked target genes. For example, an NES of 3.0 corresponds to a false discovery rate (FDR) between 0.03 and 0.09. Significant motifs were linked to TFs using *Homo sapiens* annotation databases [23].

## 3. Results

### 3.1. Synovium Sample Analysis

We analyzed synovial samples using scRNA-seq analysis from four patients with RA and five patients with PsA from GSE200815, and twelve datasets of knee OA from 51 patients of GSE176308 and three patients of GSE152805, and the synovial fluid sample of GSE161500 from three PsA patients (Figure 1A). Filtered genes were integrated with batch correction using Seurat, followed by comprehensive bioinformatics analyses of the samples. In total, approximately 5500 cells were analyzed in RA patients, 18,700 cells in PsA, 1300 cells in OA, and 250 cells in a PsA synovial fluid sample (Figure S1B). A total of 11, 12, and 10 clusters were identified in RA, PsA, and OA groups, respectively (Figure 1B–D and Figure S1C–E). There were 7 clusters identified in the PsA synovial fluid group (Figure 1E and Figure S1F). Clusters from all groups were annotated and identified based on their marker genes.

### 3.2. Antigen Presentation Process and Immune-Related Responses in Inflammatory Arthritis

In the enriched ontology clusters, we found functional groups clustering in arthritis, including the positive regulation of immune and inflammatory response, vascular development, and cell population proliferation (Figure 1F). Owing to the similar clustering patterns and GO enrichment profiles observed in the three sample sets (RA, OA, and PsA) (Figure 1G), we performed MCODE component analysis (Figure 1H) and identified the antigen processing and presentation via MHC class II, GPCR ligand binding, and downstream signaling. From this perspective, it appears that whether it is traditionally considered inflammatory arthritis, such as RA and PsA, or OA, which is generally associated with mechanical stress, all are related to immune and inflammatory responses. Moreover, they may all be initiated through an antigen presentation process involving MHC class II.

### 3.3. Fibroblast-like Synoviocytes (FLS)

In the scRNA-seq analysis of RA, PsA, and OA synovial samples, cell marker annotation revealed that FLS were the most abundant cell type across all three arthritis (Figure S1C–E,G). Based on the expression levels of specific markers, these FLS populations could be broadly categorized into three subtypes: Proteoglycan 4 (PRG4^+^), PRG4^+^MMP3^+^, and MMP3^+^. PRG4 is a mucinous glycoprotein secreted by FLS and superficial zone chondrocytes, released into the synovial fluid, and adsorbed onto cartilage and synovial surfaces to provide lubrication and protection [24]. Matrix metalloproteinases (MMPs), particularly MMP3, are a family of proteinases involved in the degradation and remodeling of the extracellular matrix (ECM). During RA, the elevation of MMP3 levels is considered a biomarker of cartilage destruction progression. Moreover, MMP3 can activate other MMPs, contributing to further ECM breakdown in articular cartilage [25].

**Figure 2:**
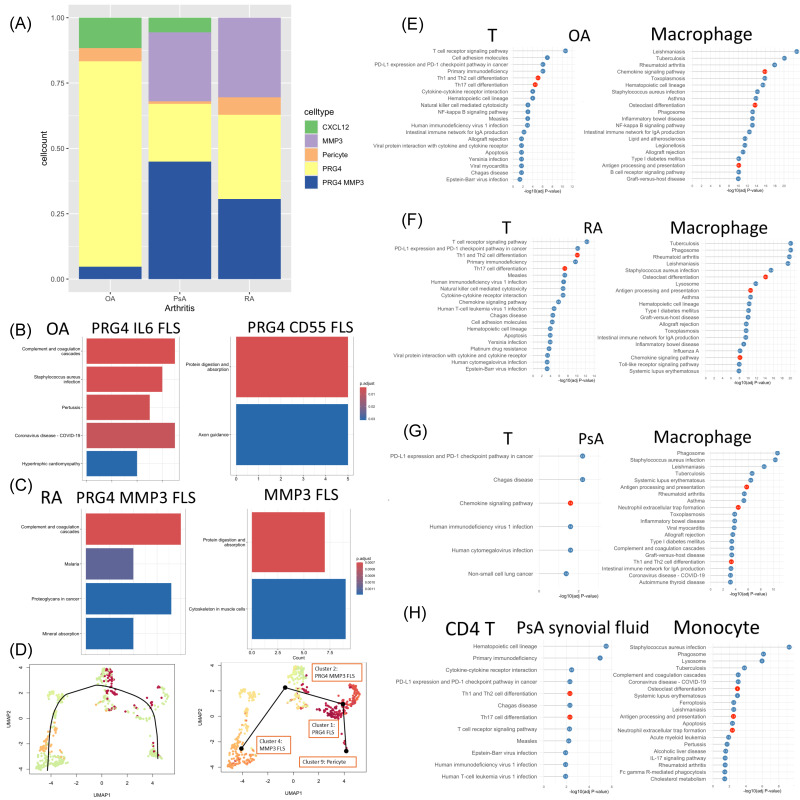
Single-cell RNA-sequencing analysis of FLS among three inflammatory arthritis. (**A**) The histogram depicts the distribution of subclusters of FLS with OA, PsA, and RA. (**B**) Bar plots showing the KEGG pathways of FLS in OA. (**C**) Bar plots showing the KEGG pathways of FLS in RA. (**D**) Suggested trajectory from pericytes, PRG4^+^ FLS, PRG4^+^MMP3^+^ FLS, and MMP3^+^ FLS of RA on the 2D map. (**E**–**G**) Lollipop chart showing the KEGG pathways of T cell and macrophage. (**E**) OA (**F**) RA (**G**) PsA. (**H**) Lollipop chart showing the KEGG pathways of CD4^+^ T cells and monocytes of the PsA synovial fluid sample.

Regarding the distribution of these three FLS subtypes, OA exhibited the highest proportion of PRG4-expressing FLS, suggesting a predominance of cells associated with lubrication and homeostatic maintenance. In RA, the numbers of PRG4^+^, PRG4^+^MMP3^+^, and MMP3^+^ FLS were approximately equal, indicating a more heterogeneous FLS population with both protective and matrix-degrading features. In contrast, PsA samples showed a higher proportion of PRG4^+^MMP3^+^ FLS, followed by MMP3^+^, and the lowest proportion of PRG4^+^ FLS, suggesting a stronger matrix-degrading phenotype and a potential shift toward more inflammatory or invasive FLS subsets (Figure 2A).

### 3.4. The KEGG Analysis of Fibroblast-like Synoviocytes in Three Inflammatory Arthritis

In OA, due to the predominance of PRG4^+^ FLS, we further categorized them into two subpopulations based on gene expression profiles: PRG4^+^IL6^+^ and PRG4^+^CD55^+^. KEGG pathway analysis revealed that PRG4^+^IL6^+^ cells were enriched in pathways associated with complement and coagulation cascades, whereas PRG4^+^CD55^+^ cells were enriched in pathways related to protein digestion and absorption (Figure 2B). In contrast, in RA, the PRG4^+^MMP3^+^ subset was associated with complement and coagulation cascades, while the MMP3^+^ FLS population showed enrichment in protein digestion and absorption (Figure 2C). Notably, in PsA, no specific FLS subpopulation demonstrated significant enrichment in either pathway.

### 3.5. The Differentiation Trajectory from Pericyte to FLS

Using slingshot trajectory analysis based on single-cell transcriptomic data from RA synovial tissue samples, we observed a dynamic differentiation process from pericyte to synovial FLS. The differentiation appears to progress from cluster 9-pericyte to cluster 1-PRG4^+^ FLS, which are primarily located in the lining layer and associated with homeostatic functions, toward more activated and matrix-degrading phenotypes, cluster 2-PRG4^+^MMP3^+^ and cluster 4-MMP3^+^ FLS (Figure 2D). This trajectory suggests a transition from protective to pathogenic role in the context of synovial inflammation. In particular, the PRG4^+^MMP3^+^ FLS may represent an intermediate or primed state that contributes to tissue remodeling and inflammation, eventually giving rise to MMP3^+^ FLS, which are involved in ECM degradation and joint destruction.

### 3.6. The KEGG Analysis of T Cells and Macrophages in Three Inflammatory Arthritis

In both OA and RA, T cells exhibited enrichment in Th1, Th2, and Th17 cell differentiation pathways, while macrophages were associated with osteoclast differentiation and antigen processing and presentation. In contrast, in PsA, T cells were enriched in the chemokine signaling pathway. Meanwhile, macrophages in PsA demonstrated enrichment in antigen processing and presentation, Th1 and Th2 cell differentiation, and neutrophil extracellular trap formation (Figure 2E–G). In PsA synovial fluid sample, CD4^+^ T cells exhibited enrichment in Th1, Th2, and Th17 cell differentiation pathways, while monocytes were associated with osteoclast differentiation, antigen processing and presentation, and neutrophil extracellular trap formation (Figure 2H).

### 3.7. The Inference of Cell–Cell Interaction (Ligand–Receptor Interaction)

**Figure 3:**
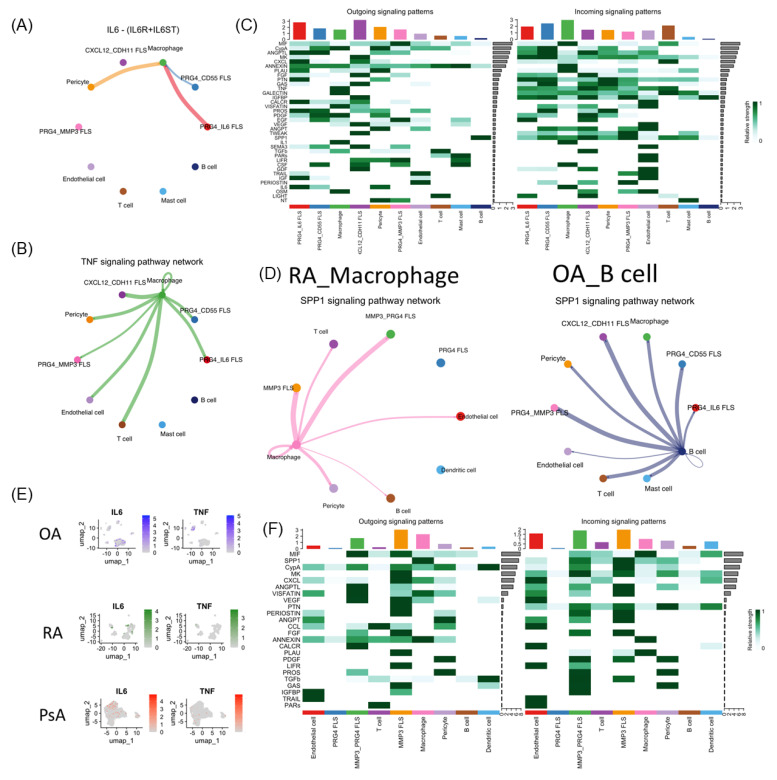
Functional differences between cell cluster interactions for candidate signaling pathways. (**A**,**B**) Plots showing cell–cell interaction and strength for a specific pathway in OA. (**A**) IL6 (**B**) TNF. (**C**) Heatmap highlighting the differential cell interaction strengths of outgoing and incoming signals of OA. (**D**) Plots showing cell–cell interaction and strength for the SPP1 pathway in RA and OA. (**E**) The cell marker expression of IL6 and TNF on the 2D map of OA, RA, and PsA. (**F**) Heatmap highlighting the differential cell interaction strengths of outgoing and incoming signals of RA.

In OA, PRG4^+^ FLS and pericytes are predicted to initiate inflammatory signaling by promoting macrophage activation through IL6 (Figure 3A). Subsequently, macrophages are inferred to influence FLS, pericytes, endothelial cells, and T cells through TNF signaling (Figure 3B). Within the FLS population, we identified a subset characterized by high CXCL12 and CDH11, but low PRG4 expression. These CXCL12^+^CDH11^+^ FLS may modulate the OA microenvironment by interacting with FLS, pericytes, endothelial cells, and B cells through IGFBP signaling (Figure 3C). In addition, B cells are predicted to contribute to the inflammatory network by engaging FLS, pericytes, T cells, and mast cells through secreted phosphoprotein 1 (SPP1) signaling (Figure 3D). Finally, mast cells may exert a regulatory effect by providing negative feedback to macrophages through annexin-mediated signaling (Figure 3C).

In RA, prominent IL6- and TNF-mediated interactions are no longer evident. IL6 cell marker expression is mainly confined to cluster 2-PRG4^+^MMP3^+^ FLS and cluster 4-MMP3^+^ FLS (Figure 3E). Instead, macrophages are predicted to act as the dominant signaling hubs, influencing multiple cell types, including FLS, pericytes, and T cells, primarily through SPP1 signaling (Figure 3D). In addition, macrophage-FLS interactions were inferred to occur via VISFATIN signaling (Figure 3F). Similarly, in PsA, IL6 and TNF signaling are not prominently detected. IL6 expression is limited to a subset of FLS in cluster-1, characterized by high MMP3 expression (Figure 3E).

**Figure 4:**
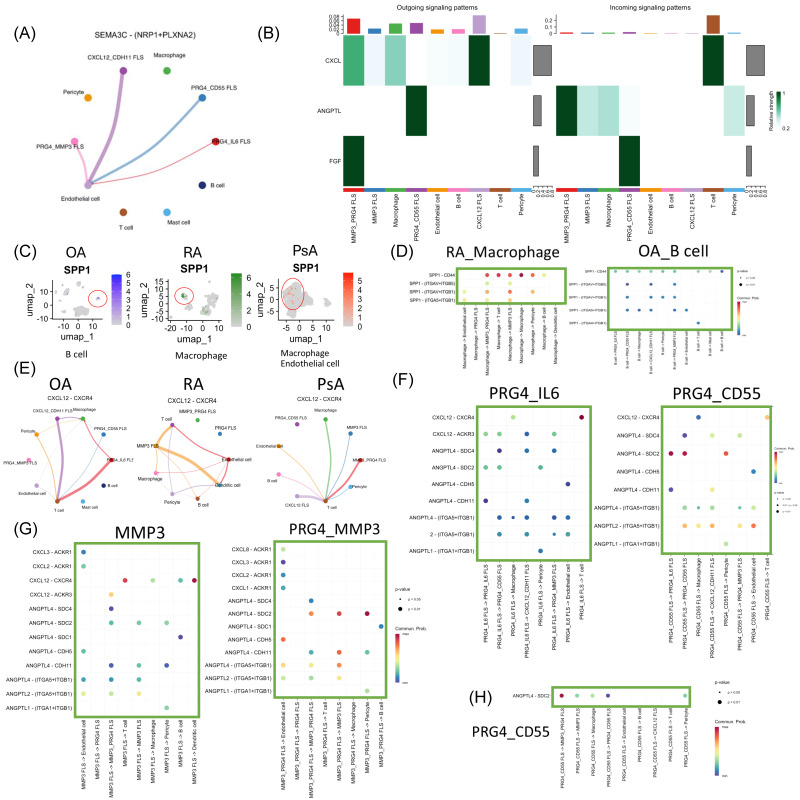
Functional differences between cell cluster interactions for candidate signaling pathways. (**A**) Plots showing cell–cell interaction and strength for the SEMA3C pathway in OA. (**B**) Heatmap highlighting the differential cell interaction strengths of outgoing and incoming signals of PsA. (**C**) The cell marker expression of SPP1 on the 2D map of OA, RA, and PsA. Red circles denote cell clusters representing B cell, macrophage, macrophage and endothelial cell, respectively. (**D**) Cell communication by ligand-receptor interaction of the SPP1 pathway in RA and OA. (**E**) Plots showing cell–cell interaction and strength for the CXCL12 pathway in OA, RA, and PsA. (**F**–**H**) Cell communication by ligand-receptor interaction of the ANGPTL pathway of FLS. (**F**) OA (**G**) RA (**H**) PsA.

Semaphorin-3c (SEMA3C) ligand with Plexin A2 (PLXNA2) and Neuropilin-1 (NRP1) can promote angiogenesis [26]. In OA, CXCL12^+^CDH11^+^ FLS and PRG4^+^CD55^+^ FLS are inferred to influence endothelial cells through SEMA3C signaling, potentially promoting angiogenesis (Figure 4A). In contrast, comparable SEMA3-mediated interactions are not detected in RA or PsA (Figure 3F and Figure 4B).

SPP1 is important in the pathogenesis of inflammatory arthritis by promoting cell proliferation and osteoclast formation via the PI3K-AKT and Janus kinase (JAK)/STAT3 signaling pathway [27,28]. In OA, SPP1 signaling is predominantly attributed to B cells, whereas in RA, this pathway is primarily mediated by macrophages (Figure 4C). Notably, SPP1 signaling intensity by macrophages in RA is substantially higher than that by B cells in OA (Figure 4D). In contrast, macrophages in OA are mainly associated with TNF-mediated signaling (Figure 3C).

This suggests that during the early stages of arthritis, PRG4^+^ FLS may initiate the inflammatory cascade by secreting IL6, which in turn stimulates macrophages to produce TNF, driving FLS activation for joint repair, angiogenesis, and inflammation. As the immune response progresses and B cells become involved, they begin to produce SPP1 to enhance proliferation and amplify the inflammatory response. However, in RA, the production of SPP1 is taken over by macrophages, with significantly greater intensity, contributing to a more robust and persistent inflammatory process compared to OA. This heightened and sustained inflammation likely underlies the more severe joint destruction observed in RA relative to OA.

In PsA, we did not detect prominent SPP1-mediated signaling from either B cells or macrophages toward FLS or other immune–inflammatory cells. Instead, inferred cell–cell communication analyses suggested that multiple cell types, including FLS, predominantly signal through CXCL12–CXCR4 interactions to activate T cells (Figure 4E).

This distinct lack of local SPP1-driven activation, coupled with the widespread CXCL12-T cell axis, may suggest that immune–inflammatory activation in PsA does not originate primarily within the joint environment, but rather reflects an external or systemic immune trigger, such as that occurring in skin or entheses, which then secondarily influences synovium. This interpretation aligns with clinical and immunopathological observations that PsA often involves enthesitis and skin inflammation (psoriasis) as early manifestations, and supports the notion that PsA synovitis may be more “infiltrative” rather than “locally initiated”, in contrast to RA.

### 3.8. T Cell Activation by FLS Subtypes Through CXCL12 Signaling

Across inflammatory arthritis subtypes, FLS appears to play a central role in sustaining immune-mediated inflammation by promoting T cell activation through CXCL12–CXCR4 signaling. In OA, this function is mainly attributed to PRG4^+^IL6^+^ FLS and CXCL12^+^CDH11^+^ FLS. In RA, CXCL12-mediated T cell activation is predominantly associated with MMP3^+^ FLS. In PsA, although multiple cell types contribute, PRG4^+^MMP3^+^ FLS and other CXCL12-high FLS represent the major inferred drivers of T cell activation (Figure 4E).

### 3.9. Cell–Cell Interactions Among Different Subsets of FLS

During the progression of arthritis, FLSs undergo a dynamic differentiation process, with distinct FLS subsets emerging at various stages. Each subset may perform specialized functions and interact with one another to shape the inflammatory microenvironment through FGF and ANGPTL signaling pathways.

Within the FGF signaling pathway, distinct interaction patterns are inferred among FLS subsets across arthritis types. In OA, PRG4^+^CD55^+^ and PRG4^+^IL6^+^ FLS exhibit reciprocal interactions mediated by FGF10. PRG4^+^MMP3^+^ FLS are predicted to influence PRG4^+^CD55^+^ and PRG4^+^IL6^+^ FLS through FGF2 and FGF18, while a CXCL12^+^CDH11^+^ FLS subset targets these PRG4^+^ populations through FGF7. Collectively, these interactions appear to converge on modulation of PRG4^+^ FLS activity (Figure S1H).

In contrast, in RA, FGF signaling appears largely independent of PRG4-dominant FLS populations. PRG4^+^MMP3^+^ FLS are inferred to interact with MMP3^+^ FLS through FGF10, whereas MMP3^+^ FLS influence PRG4^+^MMP3^+^ FLS through FGF7. In PsA, the inferred FGF network is more limited, with PRG4^+^MMP3^+^ FLS signaling to PRG4^+^CD55^+^ FLS primarily through FGF10 (Figure S1H).

Within the ANGPTL signaling pathway, ANGPTL2 and ANGPTL4 display distinct interaction patterns across arthritis subtypes. In OA, PRG4^+^IL6^+^ and PRG4^+^CD55^+^ FLS exhibit largely similar signaling profiles. ANGPTL4 is inferred to act primarily in an autocrine or paracrine manner through syndecan receptors SDC2 and SDC4 on FLS, with notable interactions through SDC2 on pericytes and PRG4^+^IL6^+^ FLS, as well as through CDH11 on PRG4^+^IL6^+^ FLS. Both ANGPTL4 and ANGPTL2 are predicted to signal through integrin α5β1 on FLS and endothelial cells. In addition, ANGPTL4 signaling is inferred through integrin α5β1 on macrophages and through CDH5 on endothelial cells. PRG4^+^MMP3^+^ FLS shows a similar ANGPTL signaling pattern, although without significant inferred interactions involving macrophages (Figure 4F).

In RA, PRG4^+^ FLS shows limited involvement in ANGPTL signaling. ANGPTL4 is inferred to form PRG4^+^MMP3^+^ and MMP3^+^ FLS, acting through SDC2, SDC4, and CDH11 on FLS and pericytes, and through CDH5 on endothelial cells. Both ANGPTL2 and ANGPTL4 are also predicted to signal through integrin α5β1 on FLS and endothelial cells (Figure 4G). In both OA and RA, ANGPTL1 interactions are inferred through integrin α1β1 on pericytes. In contrast, in PsA, ANGPTL4 signaling appears restricted to PRG4^+^CD55^+^ FLS, acting through SDC2 on FLS, pericytes, and macrophages, with no detectable interactions involving endothelial cells (Figure 4H).

### 3.10. The Immune, Inflammatory, and Angiogenic Pathway in FLS

Collectively, these analyses suggest that FLSs adopt three major functional roles within the articular microenvironment. Upon joint injury or tissue stress, a subset of FLSs is inferred to initiate inflammation through IL6 production, leading to macrophage activation and subsequent TNF or SPP1 signaling. This cascade promotes immune and endothelial cell activation, angiogenesis, and the differentiation of FLSs into MMP-high subtypes, thereby amplifying local inflammation. Concurrently, FLS proliferation driven by FGF signaling may support cartilage repair; however, activation of ANGPTL pathways (ANGPTL4/2) is also associated with angiogenesis and expansion of proteolytically active FLSs, potentially accelerating extracellular matrix degradation.

**Figure 5:**
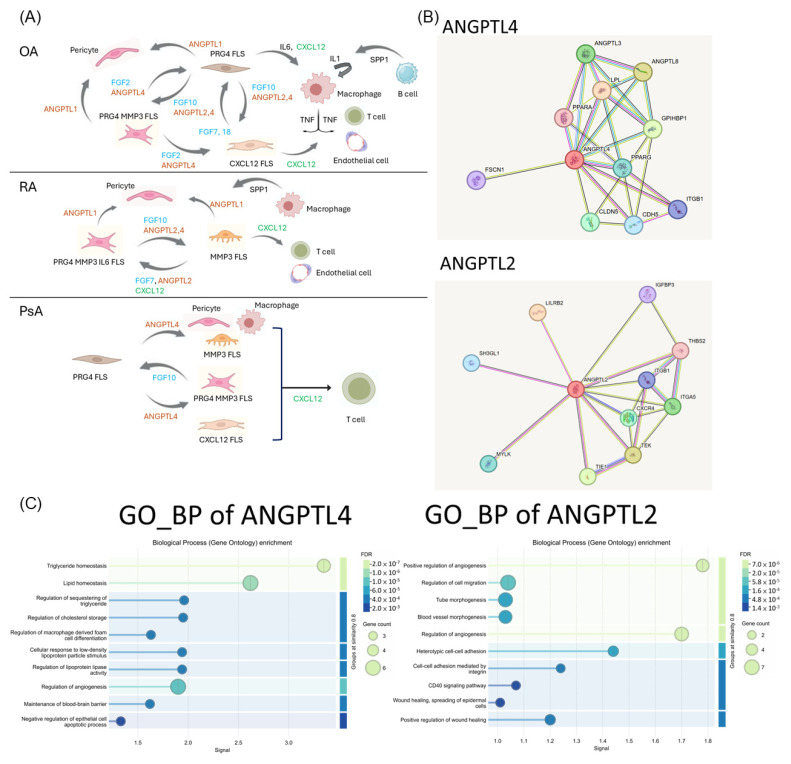
Functional differences between cell cluster interactions for three inflammatory arthritis. (**A**) Graphical abstract of the single-cell interaction map from synovium samples of OA, RA, and PsA. (**B**) The regulatory network of protein–protein interaction of ANGPTL4 and ANGPTL2. Illustrations were created using STRING. (**C**) The biological process (Gene Ontology) enrichment of ANGPTL4 and ANGPTL2.

Notably, osteoclast differentiation signatures are observed in synovial macrophages and T cells in OA and RA but are largely confined to synovial fluid monocytes and CD4^+^ T cells in PsA. These findings suggest that, unlike OA and RA, PsA-associated inflammation may be predominantly driven by immune cells recruited from the peripheral circulation following intra-articular injury, ultimately promoting osteoclast activity, MMP production, and joint destruction (Figure 5A).

### 3.11. Pathogenic Functions of ANGPTL2 and ANGPTL4 in Inflammatory Arthritis

In both OA and RA, the involvement of ANGPTL2 and ANGPTL4 is evident; however, in PsA, only ANGPTL4 appears to play a significant role. Using the STRING database, we conducted a protein–protein interaction analysis of ANGPTL2 and ANGPTL4. The results showed that ANGPTL4 is primarily associated with peroxisome proliferator-activated receptor gamma (PPARγ), CDH5, and integrin β1, whereas ANGPTL2 is more closely related to CXCR4, integrin α5, and integrin β1. These findings suggest that ANGPTL2 may have a stronger connection to immune-mediated inflammatory responses and angiogenesis (Figure 5B).

Additionally, the biological processes in GO analysis of ANGPTL4 and ANGPTL2 revealed distinct functional roles. ANGPTL4 is predominantly associated with lipid and triglyceride homeostasis, and regulation of angiogenesis, while ANGPTL2 is enriched in processes such as positive regulation of angiogenesis, cell–cell adhesion mediated by integrins, CD40 signaling pathway, and positive regulation of wound healing (Figure 5C). These findings suggest that ANGPTL2 may play a more prominent role in promoting inflammation, angiogenesis, wound healing, and cell proliferation.

### 3.12. Transcription Factor Profiling of Fibroblast-like Synoviocytes in Inflammatory Arthritis

Given the critical role of FLS in the intra-articular microenvironment, the inflammatory responses and matrix-degrading effects mediated through ANGPTL signaling and MMPs may represent key mechanisms underlying joint damage. These processes could contribute not only to the early pathogenesis of arthritis but also to the persistent joint destruction and erosive deformities observed in certain joints, even after systemic inflammation has been controlled.

We used SCENIC to identify transcription factors (TFs) that have significant regulatory effects on the key FLS involved in arthritis, specifically targeting ANGPTL2 and ANGPTL4 (Table 1). In PsA, the predominant PRG4^+^CD55^+^ FLS, with transcription factor ETS2 identified as a regulator targeting ANGPTL4. In RA, PRG4^+^MMP3^+^ FLS is the key subtype, regulated by transcription factors BCLAF1, EGR1, ELK3, and ETS1, while MMP3^+^ FLS is regulated by transcription factors ATF1, ELF1, ELF2, ELF4, ETS1, ETV3, PHF20 and RELB; all these TFs target ANGPTL2. In OA, both PRG4^+^IL6^+^ and PRG4^+^CD55^+^ FLS subtypes are relevant, with SOX4 and ZNF770 targeting ANGPTL4, while transcription factors ATF2, CREM, ELF1, ELF2, ELK3, GABPA and PHF20 target ANGPTL2 (Table 1).

Notably, in PsA, only transcription factors targeting ANGPTL4 are identified, whereas in RA, only those targeting ANGPTL2 are found. This observation suggests that ANGPTL2 and ANGPTL4 may play distinct and predominant roles in driving the progression of arthritis in RA and PsA, respectively.

## 4. Discussion

Arthritis is defined as “painful inflammation and stiffness of the joints”, including inflammatory and non-inflammatory. Inflammatory arthritis is usually associated with the classic symptoms of inflammation, pain, erythema, warmth, swelling, and loss of function [2]. In inflammatory arthritis, autoimmunity represents a broad spectrum that encompasses RA, PsA, and even OA, which was previously considered a non-inflammatory condition.

The articular capsule encloses diarthrodial joints and is lined by synovium, which produces synovial fluid to maintain cartilage lubrication and joint homeostasis. The synovium contains FLS, macrophages, and other stromal and immune cells and undergoes continuous remodeling in response to mechanical stress [29].

In RA, FLS become aberrantly activated, exhibiting increased proliferation, reduced apoptosis, and enhanced production of inflammatory mediators and MMPs. These changes drive pannus formation and transform the synovium into a site of chronic immune-mediated inflammation, ultimately leading to progressive joint destruction [30]. The synovium not only maintains the physiological state of the joint but also becomes a site of immune-mediated inflammation, further contributing to joint destruction caused by chronic inflammation [31].

The FLS in established RA synovium can play multiple roles, including the complement and coagulation systems, promotion of angiogenesis, inflammation, and degradation of cartilage and the extracellular matrix. During the progression of RA, FLSs acquire an invasive and aggressive phenotype, characterized by enhanced migratory capacity and reduced attachment-dependent growth. These changes contribute to hyperplasia of the intimal lining layer, destruction of articular cartilage, and ultimately pannus formation [30]. Based on specific cell markers, we classified FLS into three major subtypes: PRG4^+^, PRG4^+^MMP3^+^, and MMP3^+^. Further analysis of their differentiation trajectory revealed a progressive transition from pericytes to PRG4^+^ FLS, then to PRG4^+^MMP3^+^ FLS, and ultimately to MMP3^+^ FLS (Figure 2D). Since all these cell types coexist in the RA synovium, it can be inferred that pericytes and PRG4^+^ FLS are primarily responsible for maintaining joint homeostasis under normal conditions. However, in response to cartilage damage regardless of the cause, FLSs undergo differentiation to support angiogenesis and recruit immune and inflammatory responses. Although this process aims to facilitate cartilage repair, it may inadvertently exacerbate inflammation and lead to joint destruction through the increased production of MMPs.

In OA analysis, FLS exhibits a broad range of functional roles. Based on marker profiling, we identified PRG4^+^IL6^+^ and PRG4^+^CD55^+^ FLS subpopulations associated with distinct biological processes, including complement and coagulation cascades and protein digestion and absorption, respectively (Figure 2B). These findings suggest that, although OA FLS does not show a clear differentiation into an MMP3^+^ subtype as observed in RA (Figure 2C), functional diversification has already emerged within the PRG4^+^ population, with subsets contributing to joint homeostasis or inflammatory degradation.

Cell–cell interaction analyses further indicated that PRG4^+^IL6^+^ FLS may influence macrophages through IL6, followed by macrophage-mediated activation of additional FLS, endothelial cells, and T cells through TNF signaling. This inferred cascade may contribute to enhanced angiogenesis and inflammation in the OA joint microenvironment. While comparable IL6- and TNF-mediated signaling are not prominent in RA or PsA, FLS subsets expressing IL6-associated markers were still detectable (Figure 3E). Notably, OA exhibited a distinct macrophage population with strong TNF-associated signatures, which is less evident in RA and PsA (Figure 3E). This pattern suggests that, with disease progression and increasing joint damage, macrophages may shift away from TNF-driven inflammatory signaling. Such a transition could help explain why joint destruction may persist in some RA patients despite effective TNF-targeted biologic therapy [13,14].

In the joint microenvironment of OA and RA, T cells are inferred to be involved in Th1, Th2, and Th17 cell differentiation, suggesting a broad contribution to immune–inflammatory response (Figure 2E,F). In PsA, T cells primarily engage in chemokine-mediated signaling, promoting recruitment of circulating CD4^+^ T cells (Figure 2G). These recruited CD4^+^ T cells also undergo Th1, Th2, and Th17 differentiation (Figure 2H). In these three types of arthritis, FLSs are predicted to modulate T cells mainly through the CXCL12–CXCR4 axis (Figure 4E).

In RA, cell–cell interaction analysis inferred that macrophages predominantly signal through SPP1 to activate FLS, T cells, and endothelial cells (Figure 3D). In OA, macrophages show minimal SPP1 activity (Figure 4C); instead, B cells are predicted to secrete SPP1 to promote cell proliferation, although the inferred effect is substantially weaker than macrophage-derived SPP1 in RA (Figure 3D and Figure 4D). SPP1, also known as osteopontin (OPN), is a multifunctional secreted glycoprotein that regulates adhesion, proliferation, differentiation, migration, and survival in various cell types [32]. In RA, the inferred shift in macrophages toward SPP1 signaling may promote differentiation of FLS into MMP3^+^ subtypes, potentially exacerbating joint inflammation and tissue destruction more than in OA. In contrast, macrophage-derived TNF and B cell–derived SPP1 in OA are predicted to have a limited effect on FLS differentiation, which may contribute to the comparatively milder inflammation and joint damage.

Bone and cartilage are highly related to each other, as most bones develop through endochondral ossification. In this process, cartilage primordia formed during early development are gradually replaced by bone at the center, while cartilage located at the ends remains as articular cartilage. Postnatally, bone and cartilage perform distinct but complementary functions. Bone undergoes continuous remodeling through the coordination of bone resorption and formation by osteoclasts and osteoblasts, ensuring proper skeletal shape, strength, and mobility. Articular cartilage consists of chondrocytes embedded in a dense extracellular matrix, providing lubrication and shock absorption to facilitate smooth joint movement [33].

In the microenvironment of arthritis, FLS primarily interact with each other through FGF signaling. In our analysis, FGF2, FGF7, FGF10, and FGF18 were inferred as the primary ligands. Tyrosine phosphorylation of the intracellular domain of FGF receptors (FGFRs) activates multiple downstream signaling cascades, the Ras-mitogen-activated protein kinase (MAPK) pathways, including extracellular signal-regulated kinase (ERK)1/2, p38-MAPK, and c-JunN-terminal kinase (JNK), the phosphatidylinositol 3-kinase-(PI3 K-) Akt pathway, and the phospholipase C (PLC)*γ*-protein kinase C (PKC) pathway [34]. The functions of the FGF family members in arthritis are diverse. FGF2 primarily promotes osteoblastic cell proliferation and new bone formation. FGF18 reduces osteogenic cells and osteoblast maturation, matrix mineralization, and increases cell proliferation. FGF7 contributes to the formation of mineralized nodules and the expression of osteoblast marker genes [35]. Another study reported that FGF7 exerts functions including promoting cartilage destruction, inducing subchondral bone remodeling, and triggering premature growth plate closure [36]. Additionally, FGF10 has been shown to inhibit TGF-β-induced fibrosis in FLSs by downregulating the IL6/JAK2/STAT3 signaling pathway, thereby potentially delaying the progression of OA [37]. SPP1 may promote fibrosis by enhancing TGF-β signaling through MMP9, a protease that releases latent TGF-β from its inactive complex [38,39]. FGF10 appears to counterbalance the fibrosis induced by TGF-β. Once fibrosis occurs in the synovium, even if new bone formation takes place, the fibrotic changes can lead to a loss of synovial function, ultimately disrupting the normal homeostasis of the joint.

In OA, PRG4^+^MMP3^+^ FLS is inferred to influence PRG4^+^IL6^+^ and PRG4^+^CD55^+^ FLS via FGF2 signaling, potentially contributing to bone formation. PRG4^+^IL6^+^ and PRG4^+^CD55^+^ FLS are predicted to signal through FGF10 toward CXCL12^+^CDH11^+^ FLS, which may promote angiogenesis and T cell–mediated immune responses. Additionally, CXCL12^+^CDH11^+^ FLS is inferred to act on PRG4^+^IL6^+^ and PRG4^+^CD55^+^ FLS via FGF7 and FGF18 (Figure 5A and Figure S1H). Given that PRG4^+^IL6^+^ FLS is associated with complement and coagulation cascades and PRG4^+^CD55^+^ FLS with protein digestion and absorption, this FGF signaling network may support bone mineralization while potentially limiting fibrosis. Whether this balance correlates with OA progression remains to be determined.

In RA, PRG4^+^MMP3^+^ FLS is inferred to signal via FGF10 to MMP3^+^ FLS, potentially limiting FLS-associated fibrosis. Conversely, MMP3^+^ FLS is predicted to influence PRG4^+^MMP3^+^ FLS through FGF7, which may promote cartilage degradation and subchondral bone remodeling (Figure 5A and Figure S1H). This imbalance could contribute to the joint deformities often observed in RA, where tissue homeostasis is disrupted, and cartilage repair is impaired. In PsA, the inferred FGF network is more limited, with PRG4^+^MMP3^+^ FLS signaling via FGF10 to PRG4^+^CD55^+^ FLS (Figure 4F and Figure 5A), potentially promoting extracellular matrix digestion while restraining fibrosis.

Due to the complex interactions of various FGF family members among FLS subtypes, some of which are related to bone formation, it remains unclear whether these factors directly contribute to ongoing joint destruction and deformity. Although previous studies have explored FGFs as potential therapeutic targets, there is still no clear evidence demonstrating their effectiveness in treating inflammatory arthritis [40].

In RA, hypoxia and pro-inflammatory mediators in the synovial microenvironment activate key signaling pathways in FLSs, including MAPK, PI3K/Akt, JAK/STAT, and HIF-1α. Activated RA FLSs acquire cancer-like properties, displaying locally invasive and destructive behavior within inflamed synovial tissue [30]. Semaphorins function as signaling ligands that regulate the morphology and motility of cells within the cardiovascular and immune systems [41]. Class 3 semaphorin genes, which exert inhibitory effects on cancer cell proliferation, migration, and invasion, were markedly downregulated in the synovium of patients with RA [42]. In OA, CXCL12^+^CDH11^+^ and PRG4^+^CD55^+^ FLS are inferred to influence endothelial cells through SEMA3C signaling, potentially promoting angiogenesis (Figure 4A). This SEMA3-mediated effect is not observed in RA or PsA. These findings suggest that in established RA, SEMA3-driven angiogenesis may no longer be a major contributor to disease progression, whereas in PsA, angiogenesis may be primarily driven by T cell–mediated immune responses originating from outside the joint rather than by FLS within the joint microenvironment.

ANGPTL4, also known as fasting-induced adipose factor, PPARγ angiopoietin-related protein, and hepatic angiopoietin-related protein, is a novel adipocytokine that is upregulated by fasting, PPARγ, and hypoxia, and plays a key role in regulating angiogenesis. In a previous study, hypoxia may upregulate the expression of ANGPTL4, which can enhance the production of MMP1 and MMP3 [43]. ANGPTL2 may promote Ik-B degradation, thereby inducing expression of inflammation-related NF-kB target genes. ANGPTL2 has been shown to enhance the expression and activity of MMPs through integrin α5β1-mediated activation of p38-MAPK [44].

Our analysis revealed the functional roles for ANGPTL2 and ANGPTL4 in the pathogenesis of distinct arthritis. In OA, ANGPTL4 is inferred to play a prominent role, particularly via PRG4^+^CD55^+^ and PRG4^+^IL6^+^ FLS, affecting pericytes and other FLS subsets through SDC2/4 and CDH11 receptors (Figure 5A). ANGPTL4 is also predicted to interact with endothelial cells and macrophages via integrins and CDH5, potentially promoting angiogenesis and inflammation (Figure 4F). In contrast, ANGPTL2 in OA appears primarily involved in ECM remodeling through integrin-mediated signaling.

In RA, ANGPTL2 is inferred to dominate the ANGPTL signaling network, particularly via PRG4^+^MMP3^+^ and MMP3^+^ FLS, with key transcription factors including EGR1 and ETS1 (Table 1). EGR1 can be induced by inflammatory stimuli (such as cytokines) to regulate genes involved in synovial pannus formation and joint destruction [45]. ETS1-dependent transcription was important for ANGPTL2-induced CXCR4 expression, which subsequently drives cell motility, invasion, and vascular abnormalities [46].

ANGPTL2 is predicted to act through integrin-mediated pathways (Figure 4G), notably targeting endothelial cells and potentially sustaining a more persistent and destructive inflammatory response. However, the functional significance of ANGPTL4 appears to be limited, and no definitive transcription factor targets regulating its expression could be identified. A previous study on late-stage RA reported that ANGPTL4 mRNA levels in synovial tissues were approximately 1/100 of those of ANGPTL2, suggesting that ANGPTL4 expression may be relatively higher in the early stage of RA [45].

In PsA, ANGPTL4 is inferred to be the primary mediator, particularly in PRG4^+^CD55^+^ FLS, with transcription factor ETS2 predicted to regulate its expression (Table 1). ETS2 acts as a transcriptional regulator of ANGPTL4 and induces proinflammatory responses, while ANGPTL4 modulates lipid metabolism and angiogenesis [47]. In OA, ANGPTL4 is inferred to be associated with the transcription factor SOX4, which may modulate the CXCL12–CXCR4 axis in endothelial cells and thereby contribute to neovascularization [48].

ANGPTL4 is predicted to act through SDC2 on pericytes, macrophages, and FLS, but appeared to have limited influence on endothelial cells (Figure 4H). Compared with OA and RA, PsA shows relatively minimal ANGPTL2 activity. Overall, these findings suggest that ANGPTL4 may play a prominent role in OA and PsA, potentially contributing to early inflammatory responses, angiogenesis, and tissue remodeling, whereas ANGPTL2 is more relevant in RA, supporting chronic inflammation and joint destruction.

There are several limitations to this study. First, public scRNA-seq datasets are inherently limited by small patient numbers and inter-dataset heterogeneity. These constraints are widely recognized in single-cell transcriptomic studies and necessitate cautious interpretation of computationally inferred results. Second, owing to the lack of available transcriptomic data from patients with early RA, it remains unclear whether the observed shift in SPP1 signaling, from B cell–associated patterns in OA to macrophage-associated patterns in RA, reflects a continuous pathological evolution or fundamentally distinct disease mechanisms. Consequently, additional functional validation, particularly at the cellular level, is required to assess the effects of these candidate compounds on ANGPTL-related signaling and to clarify their potential relevance in modulating FLS-associated joint pathology.

## 5. Conclusions

Within the joint microenvironment, FLS exhibits functional plasticity associated with differentiation states, suggesting their involvement in both tissue homeostasis and inflammatory responses. FLSs are also implicated in interactions with T cells and macrophages, potentially amplifying immune-mediated inflammation. In OA and RA, intercellular communication among FLS appears to be associated with FGF and ANGPTL signaling pathways. RA is further characterized by enhanced macrophage-derived SPP1 signaling, with late-stage RA showing increased ANGPTL2-associated signaling patterns, which may be linked to more pronounced joint damage. In PsA, FLS-associated signals are predominantly observed during early joint injury, potentially contributing to the recruitment of T cells and circulating immune cells through chemotactic pathways. In contrast, inflammatory responses in PsA appear to be largely driven by immune cells of hematogenous origin. The therapies for PsA may focus on systemic immune modulation and preservation of joint structural integrity. Together, these observations suggest distinct stromal–immune interaction patterns across arthritis subtypes. Overall, our findings provide an exploratory framework indicating disease-specific associations of ANGPTL-related signaling with FLS activity. While these pathways may represent potential therapeutic targets, further functional and experimental validation is required to establish their mechanistic roles in the pathogenesis of arthritis.

## Figures and Tables

**Table 1:** The transcription factors of the ANGPTL signaling pathway in FLS of arthritis.

Arthritis/FLS	TF	Gene	nMotifs	BestMotif	NES	MotifDb	Coex Module	SpearCor	CoexWeight
PsA/PRG4^+^CD55^+^	ETS2	ANGPTL4	1	tfdimers__MD00116	3.75	10 kb	top3sd	0.197902	0.016256
RA/PRG4^+^MMP3^+^	BCLAF1	ANGPTL2	1	taipale_tf_pairs__ETS2	3.1	10 kb	w0.001	0.043095	0.002935
	EGR1	ANGPTL2	1	taipale_tf_pairs__ETS2	3.33	10 kb	w0.001	0.091679	0.005957
	ELK3	ANGPTL2	2	taipale__ELK3_DBD	4.5	10 kb	w0.001	0.194195	0.005913
	ETS1	ANGPTL2	1	flyfactorsurvey__Ets65A	3.24	10 kb	w0.001	0.060482	0.00359
RA/MMP3^+^	ATF1	ANGPTL2	2	jaspar__MA0604.1	3.41	500 bp	w0.001	0.0857	0.001038
	ELF1	ANGPTL2	2	taipale_tf_pairs__E2F1	3.28	10 kb	top10	0.255602	0.01733
	ELF2	ANGPTL2	1	taipale_tf_pairs__ETS2	4.33	10 kb	w0.001	0.139592	0.002562
	ELF4	ANGPTL2	1	taipale_tf_pairs__ETS2	3.68	10 kb	w0.001	0.06648	0.001156
	ETS1	ANGPTL2	3	taipale__ETS1_DBD	3.32	10 kb	w0.001	0.125608	0.003877
	ETV3	ANGPTL2	1	taipale_tf_pairs__ETS2	3.29	10 kb	w0.001	0.040665	0.001224
	PHF20	ANGPTL2	1	taipale_tf_pairs__ETS2	4.65	10 kb	w0.001	0.043007	0.001398
	RELB	ANGPTL2	1	stark__MRYTTCCGYY	3.17	500 bp	w0.001	0.07625	0.001109
OA/PRG4^+^IL6^+^ and CD55^+^	SOX4	ANGPTL4	1	tfdimers__MD00558	3.06	10 kb	top50	0.044989	0.004093
	ZNF770	ANGPTL4	1	flyfactorsurvey_hb_FlyReg	3.13	10 kb	w0.001	0.046397	0.001287
	ATF2	ANGPTL2	1	transfac_pro__M01861	3.31	500 bp	w0.001	0.094	0.001531
	CREM	ANGPTL2	1	transfac_pro__M07682	3.66	10 kb	w0.001	0.039113	0.002271
	ELF1	ANGPTL2	2	taipale_tf_pairs__ETS2	4.75	10 kb	w0.001	0.127312	0.004881
	ELF2	ANGPTL2	1	taipale_tf_pairs__ETS2	3.58	10 kb	w0.001	0.061485	0.002499
	ELK3	ANGPTL2	1	taipale__ELK3_DBD	4.67	10 kb	w0.001	0.048437	0.001697
	GABPA	ANGPTL2	4	transfac_pro__M08917	4.07	500 bp	w0.001	0.045614	0.001261
	PHF20	ANGPTL2	1	taipale_tf_pairs__ETS2	5.4	10 kb	w0.001	0.102481	0.00339

TF: transcription factor; coexModule: co-expression module; w0.001: in each TF, retain genes with weight > 0.001 to form modules. top3sd: retain targets in each TF where the weight is greater than mean (weight) + 3 × sd (weight). top10: retain the top 10 TFs based on weight for each gene to obtain a simplified TF-target pairs list. Then, assign genes to TFs to construct co-expression modules. top50: retain the top 50 TFs based on weight for each gene to obtain a simplified TF-target pairs list. Then, assign genes to TFs to construct co-expression modules.

## Data Availability

The authors confirm that the data supporting the findings of this study are available in the article and its Supplementary Materials.

## Supplementary Materials

The following supporting information can be downloaded at: https://www.mdpi.com/article/10.3390/biomedicines14020396/s1. Figure S1: Single-cell RNA-sequencing analysis of synovium samples from osteoarthritis (OA), rheumatoid arthritis (RA), psoriatic arthritis (PsA) and PsA synovial fluid (PsASF).

## Abbreviations

ANGPTL	Angiopoietin-like protein
AUC	Area under the curve
CCP	cyclic citrullinated peptide
DEGs	Differentially expressed genes
ECM	Extracellular matrix
FDR	False Discovery Rate
FLS	Fibroblast-like synoviocytes
FGF	Fibroblast growth factor
GEO	Gene Expression Omnibus
GO	Gene Ontology
HIF-1α	Hypoxia inducible factor-1α
JAK	Janus kinase
KEGG	Kyoto Encyclopedia of Genes and Genomes
MAPK	Mitogen-activated kinase phosphatase
MMP	matrix metalloproteinases
NES	Normalized Enrichment Score
NRP1	Neuropilin-1
OPN	Osteopontin
OA	osteoarthritis
PCs	Principle components
PsA	psoriatic arthritis
PPARγ	Peroxisome proliferator-activated receptor gamma
PRG4	Proteoglycan 4
PLXNA2	Plexin A2
PI3K-Akt pathway	phosphatidylinositol 3-kinase (PI3K)-serine-threonine protein kinase (Akt) pathway
ROS	Reactive oxygen species
RA	Rheumatoid arthritis
scRNA-seq	Single-cell RNA-sequencing
SDC	syndecan receptor
SNN	Shared nearest neighbor
SCENIC	Single-cell regulatory network inference and clustering
SEMA3C	Semaphorin-3c
SPP1	Secreted phosphoprotein-1
TF	Transcription factor
UMAP	Uniform manifold approximation and projection
