# Supplementary material for: Fibroblast-like Synoviocytes as Key Regulators of Homeostasis and Inflammation in the Joint Microenvironment of Inflammatory Arthritis
Source: Biomedicines. 2026 Feb 9;14(2):396. doi: 10.3390/biomedicines14020396 (PMC12938744; doi:10.3390/biomedicines14020396)

Supplementary Figure S1. Single-cell RNA-sequencing analysis of synovium samples from osteoarthritis (OA), rheumatoid arthritis (RA), psoriatic arthritis (PsA) and PsA synovial fluid (PsASF).

(A) A ranking of principle components based on the percentage of variance of "Elbow plot".

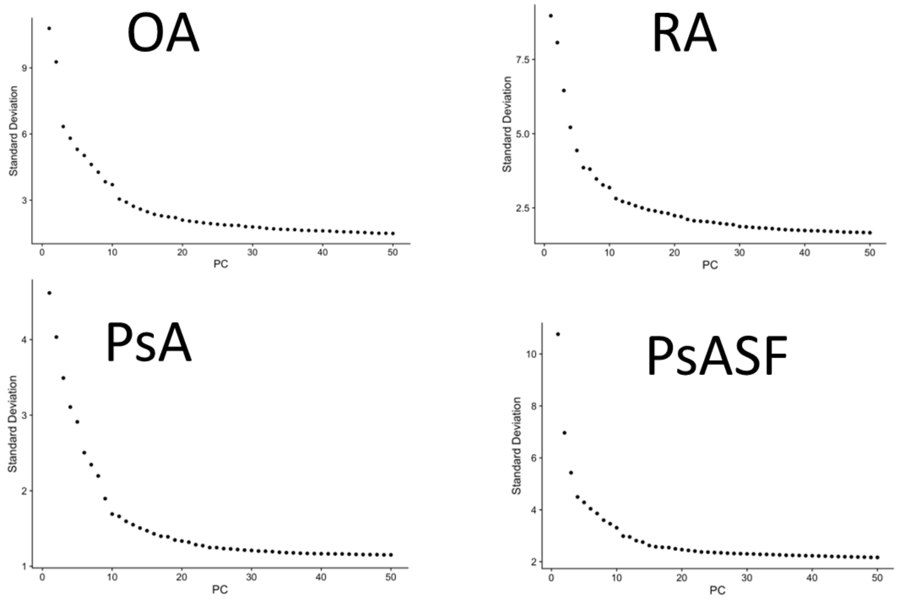

(B) The analyzed cell counts of the OA, PsA, PsASF, and RA.

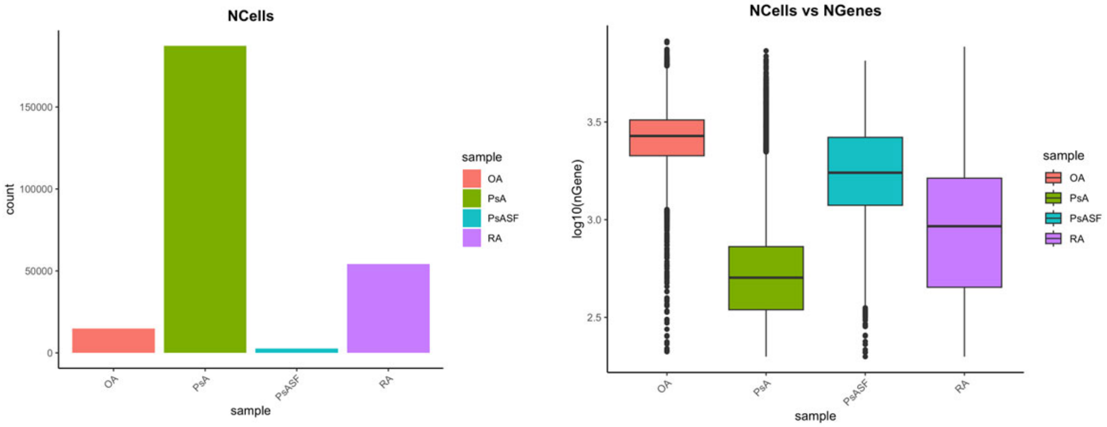

(C–F) Overview of the scRNAseq landscape. Markers were used to identify the clusters and differences of UMAP.

(C) RA

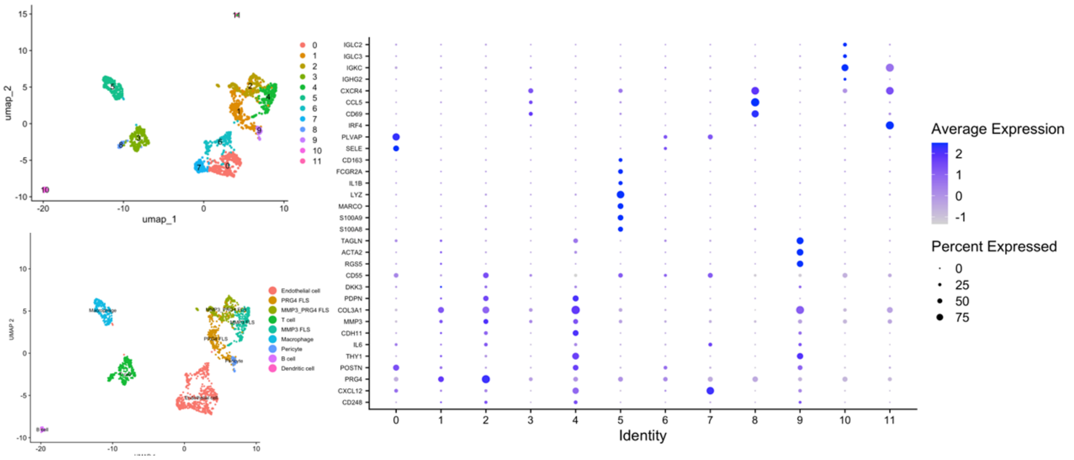

(D) PsA

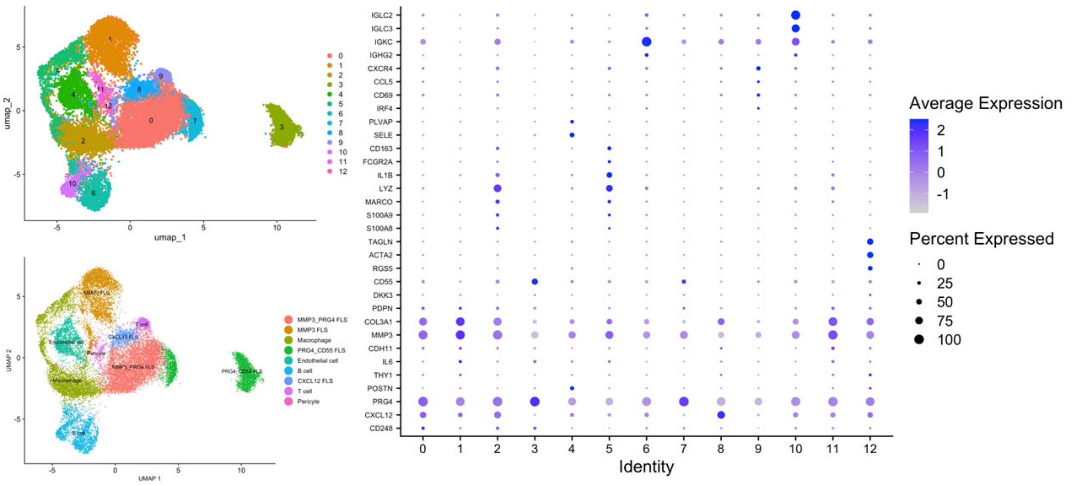

(E) OA

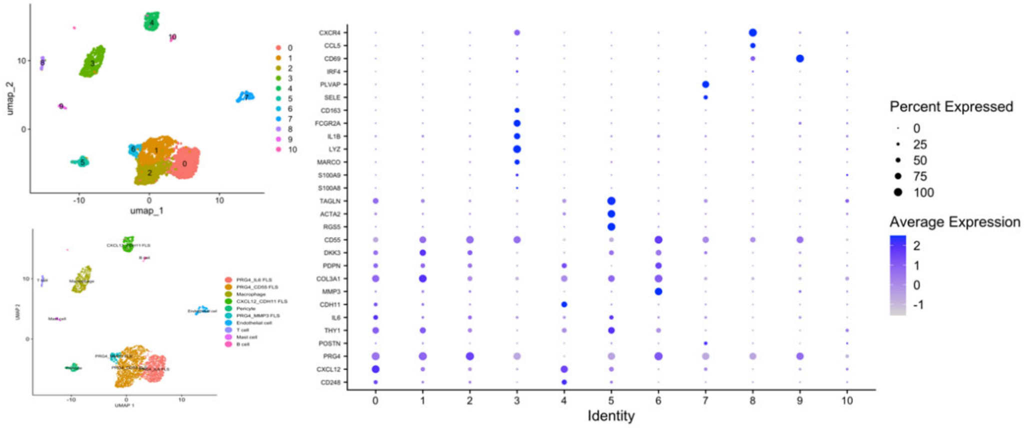

(F) PsA synovial fluid

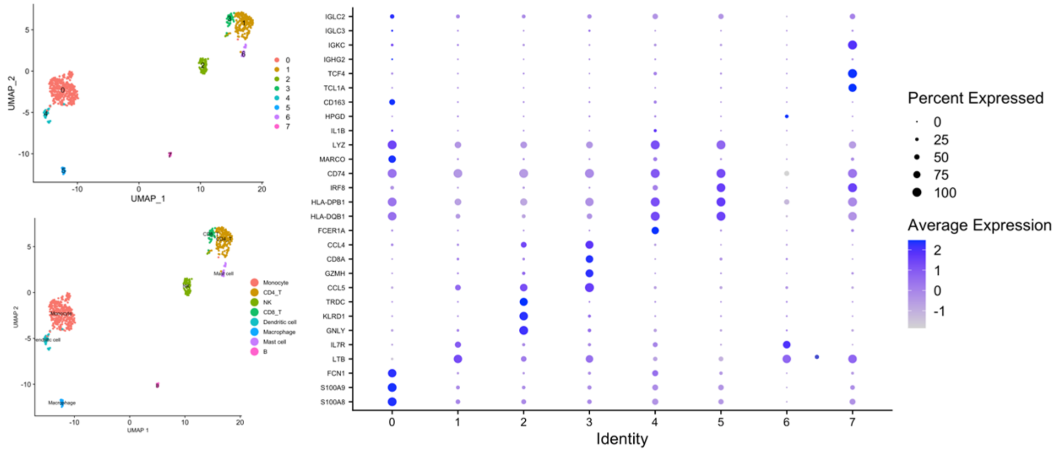

(G)The cell marker expression of IL6, TNF, ANGPTL2, ANGPTL4, PRG4 and MMP3 on the 2D map of OA, RA, and PsA.

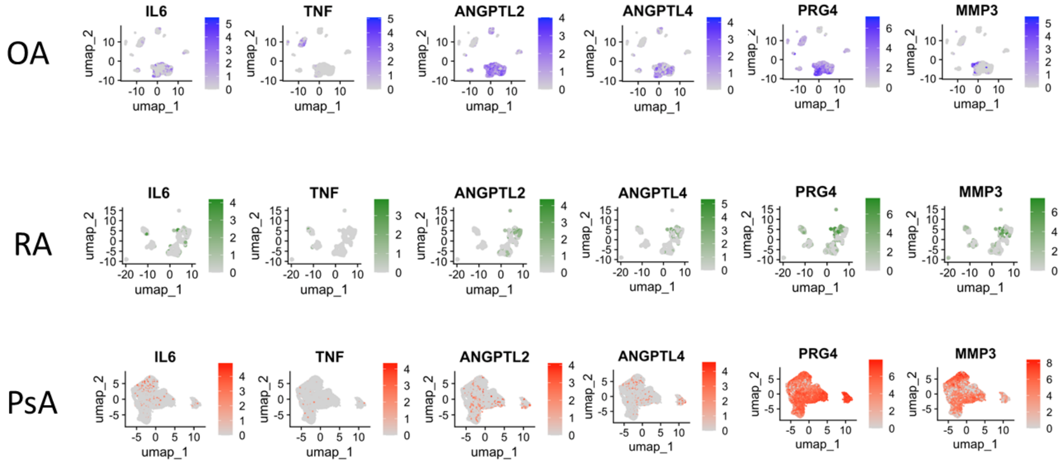

(H) Plots showing cell–cell interaction and strength for FGF pathways in OA, RA and PsA.

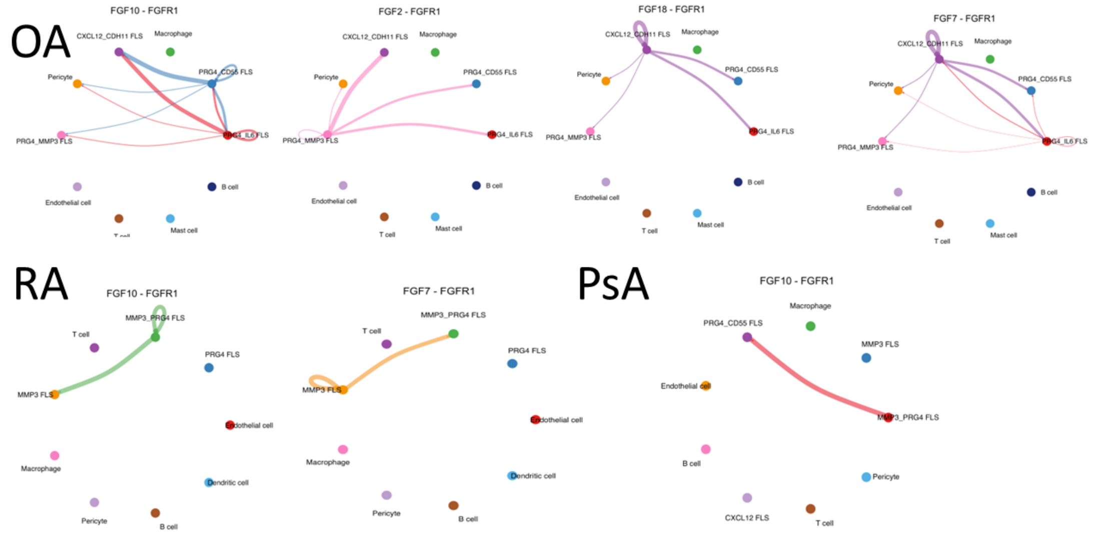

Supplement: Supplementary file 1 [file biomedicines-14-00396-s001.zip › Figure_S1.pdf]
